# The functions and mechanisms of RNA modification in prostate: Current status and future perspectives

**DOI:** 10.3389/fgene.2024.1380746

**Published:** 2024-05-10

**Authors:** Zhijin Zhang, Ji Liu, Yang Wu, Zhuoran Gu, Libin Zou, Yingdi Liu, Jiang Geng, Shiyu Mao, Ming Luo, Changcheng Guo, Wentao Zhang, Xudong Yao

**Affiliations:** ^1^ Department of Urology, Shanghai Tenth People’s Hospital, School of Medicine, Tongji University, Shanghai, China; ^2^ Urologic Cancer Institute, School of Medicine, Tongji University, Shanghai, China; ^3^ School of Medicine, Tongji University, Shanghai, China; ^4^ Department of Pathology, Shanghai Tenth People’s Hospital, Shanghai, China

**Keywords:** RNA modification, prostate cancer, AR, tumor microenvironment, CRPC

## Abstract

The increasing incidence and mortality of prostate cancer worldwide significantly impact the life span of male patients, emphasizing the urgency of understanding its pathogenic mechanism and associated molecular changes that regulate tumor progression for effective prevention and treatment. RNA modification, an important post-transcriptional regulatory process, profoundly influences tumor cell growth and metabolism, shaping cell fate. Over 170 RNA modification methods are known, with prominent research focusing on N6-methyladenosine, N7-methylguanosine, N1-methyladenosine, 5-methylcytidine, pseudouridine, and N4-acetylcytidine modifications. These alterations intricately regulate coding and non-coding RNA post-transcriptionally, affecting the stability of RNA and protein expression levels. This article delves into the latest advancements and challenges associated with various RNA modifications in prostate cancer tumor cells, tumor microenvironment, and core signaling molecule androgen receptors. It aims to provide new research targets and avenues for molecular diagnosis, treatment strategies, and improvement of the prognosis in prostate cancer.

## 1 Introduction

Prostate cancer (PCa) is one the most common cancers in men, accounting for one-third of all male malignant tumors. In 2022, it ranked highest among male tumors in the United States (Siegel et al., 2023), while in China, its incidence and mortality are increasing annually due to lifestyle changes (Chen et al., 2016). The primary treatment for PCa is androgen deprivation therapy (ADT). However, as the tumor progresses, most patients advance to the castration-resistant PCa (CRPC) stage (Prostate cancer, 2021), with a poor prognosis. Although androgen-receptor pathway inhibitors (ARPIs), such as abiraterone, enzalutamide, and apalutamide are available at this stage, their effectiveness typically lasts only approximately 18 months, posing challenges for subsequent treatment (Yan, 2022; Bergengren et al., 2023).

The emergence of epigenetics has revolutionized our understanding of cancer (Hanahan, 2022) revealing that tumor development is not solely driven by genetic changes but by modifications to genetic information such as chromatin DNA methylation and histone modification (Ge et al., 2022). Furthermore, Hechuan et al. were the first to uncover reversible methylation modifications of RNA, demonstrating that RNA is also involved in the regulation of gene expression (Frye et al., 2018). Various RNA modification methods including N6-methyladenosine (m6A), N7-methylguanosine (m7G), 5-methylcytidine (m5C), and N4-acetylcytidine (ac4C) can affect both coding and non-coding RNA species. The latter comprise micro RNA (miRNA), long non-coding RNA (lncRNA), circular RNA (circRNA), transfer RNA (tRNA), and ribosomal RNA (rRNA) (Hu et al., 2022). RNA modification, an important post-transcriptional mechanism, influences essential biological processes, providing novel avenues for exploring the mechanisms underlying tumor progression and novel therapeutic targets.

Multiple reliable sequencing results have confirmed that prostate cancer is highly heterogeneous between primary and metastatic foci. In addition, the remarkable multifocality of prostate cancer has been confirmed to belong to non-overlapping evolutionary trajectories (Løvf et al., 2019). Multiple types of high-throughput sequencing have suggested that there are significant differences in RNA modification enzymes that regulate epigenetic inheritance (Spratt et al., 2016). This also indicates that RNA modification has a potential impact on the occurrence and progression of PCa.

This review comprehensively summarizes the importance of various RNA modifications in diagnosing, and understanding the occurrence and progression of PCa, as well as their role in molecular targeted therapy for the disease.

## 2 Overview of RNA modification

### 2.1 m6A

m6A denotes the methylation of the sixth N atom of adenine within RNA molecules. It is widespread across cell types, with modification sites typically found in the 3′-untranslated region (3′UTR), near stop codons, long inner exon, intergenic regions, introns, and 5′UTR (Zhuang et al., 2023). The m6A modification process is reversible and mainly regulated by m6A RNA methylation regulators, which include over 20 identified enzymes as “writers”, “erasers”, and “readers”. “Writer” refers to m6A methyltransferases including Wilms tumor 1-related protein (WTAP) (Ping et al., 2014), zinc finger CCCH domain-containing protein 13 (ZC3H13), KIAA1429, methyltransferase-like 3 (METTL3), METTL14, and RNA binding motif protein 15 (RB M15). “Eraser” refers to demethylases such as fat mass and obesity-associated protein (FTO), alkB homolog 3 (ALKBH3), and ALKBH5. “Readers” serve as binding proteins and include YTH domain-containing 1 (YTHDC1), YTHDC2, N6-methyladenosine RNA-binding protein 1 (YTHDF1), YTHDF2, YTHDF3, insulin-like growth factor 2 mRNA-binding protein 1 (IGF2BP1), IGF2BP2, IGF2BP3, heterogeneous nuclear ribonucleoprotein C(HNRNPC), heterogeneous nuclear ribonucleoprotein A2/B1 (HNRNPA2B1), and RNA-binding motif protein X-linkage (RBMX).

The m6A modification significantly impacts the post-transcriptional regulation of RNA. Firstly, it can alter the local secondary structure of RNA and reversibly regulate RNA-protein binding motifs, affecting RNA-protein interactions (An and Duan, 2022). Secondly, m6A modification regulates the translation of mRNA through various mechanisms such as promoting mRNA cyclization and ribosome binding. YTHDF proteins are the main m6A-binding proteins in the cytoplasm (Liao et al., 2022). They can recruit heat-responsive protein 12-RNase for mitochondrial RNA processing complex, carbon catabolite repression-negative on TATA-less deenylase complex, and interact with UPF1, playing a key role in mRNA degradation. Additionally, m6A modification is closely related to chromatin accessibility, histone modification, and homologous recombination repair (Zhu et al., 2023). However, the role of these complex regulatory mechanisms in determining the fate of tumor cells is often contradictory due to differences in tumor types, warranting further investigation. For example, ALKBH5 has been shown to be involved in the malignant progression of tumors in breast cancer, gastric cancer, and glioblastoma, and its expression is significantly associated with poor prognosis (Jiang Y. et al., 2020). In pancreatic cancer, the loss of ALKBH5 affects the methylation of lncRNA KCNK15-AS1 and promotes tumor cell migration (He et al., 2018). Similarly, in pancreatic cancer, ALKBH5 has been shown to inhibit tumor progression by affecting the Wnt signaling pathway (Bai et al., 2019). In addition, YTHDF2 has also been shown to have completely opposite effects in different tumors. It inhibits tumor development by promoting the translation of the tumor suppressor *HINT2* in melanoma (Jia et al., 2019). However, highly expressed YTHDF2 in lung cancer affects tumor malignant progression by promoting the stability of *6PGD* mRNA (Sheng et al., 2020). All these suggest that RNA modifications may play opposing roles in different cell fates.

### 2.2 m7G

m7G modification predominantly occurs in the 5′cap, 5′UTR, and A-G rich regions of mRNA. The methyltransferase complex RNMT/RAM catalyzes the addition of methyl groups from S-adenosyl methionine to the N7 position of guanine nucleotide, forming the “cap” structure of m5G (5′) ppp (7′) X (Blersch et al., 2021). m7G modification of tRNA occurs at the G46 position, while in rRNA, it occurs at position G1639 of the 18S rRNA 3′ main domain.

Complexes involving METTL1 and WD repeat domain 4 (WDR4) as well as Williams-Beuren syndrome chromosome region 22 and tRNA methyltransferase activator subunit 11-2 (TRMT112) catalyze these modifications. m7G modification exerts significant regulatory effects in both normal and tumor cells (Cui et al., 2022). It is involved in regulating the stemness differentiation of embryonic cells, promoting angiogenesis, and cell self-renewal. In tumor cells, the METTL1 complex and eukaryotic translation initiation factor eIF4E often affect the stability of mRNA, promoting proliferation, migration, and drug resistance in various tumors (Hong et al., 2023).

### 2.3 m5C

m5C, first reported in 1925, is found in animals and plants and involves the addition of a methyl group to the cytosine ring. Similar to m7G, m5C is mainly enriched in the GC-rich sequence of the 3 ′UTR and around the start codon (Song et al., 2022). These regions play crucial roles in regulating mRNA stability, nuclear export, translation, and DNA damage repair. In tRNA, m5C is mainly located at positions 34, 38, 47-50, and 72, concentrated between the anticodon loop, variable loop, T₂C loop, and near the amino acid acceptor stem.

This modification helps maintain the stability of tRNA, promotes translation, and improves translation accuracy. In lncRNA, m5C modification is associated with ribosome maturation and translation efficiency (Hoernes et al., 2016). The overall methylation level of m5C is estimated at 0.02% (Dai et al., 2021), and its modification level is regulated by three enzymes: methyltransferases like NSUN1-7 and DNMT2, which add the active methyl group to the carbon at position 5 of the cytosine base in RNA; demethylases including alpha-ketoglutarate-dependent dioxygenase (ABH1) and ten-eleven translation family proteins (TET), which remove m5C modification on RNA; and readers such as RNA and export factor binding protein 2 (ALYREF) and Y box binding protein 1 (YBX1), which recognize m5C in RNA. The NSUN family consists of seven members (NSUN1-7), each targeting a different type of RNA. NSUN1, 4, and 5 methylate rRNA, while NSUN2, 3, and 6 methylate tRNA. Furthermore, NSUN2 also promotes mRNA methylation, and NUSN7 is responsible for enhancer RNA (eRNA) methylation (Sun et al., 2022).

### 2.4 N1-methyladenosine (m1A)

m1A modification is a highly conserved RNA modification, typically occurring in the GC-rich region of the first exon of mRNA (Song et al., 2021). These modifications can disrupt Watson-Crick base pairing, altering the secondary/tertiary structure of the 5′UTR in mRNA. In tRNA, m1A is stably expressed and, at positions 58 and 9, is essential for forming the clover structure. In addition, rRNA modified with m1A at position 1,322 affects the formation of 60S ribosomes (Li D. et al., 2022). “Writers” for m1A modification include TRMT6, TRMT61A, TRMT61B, TRMT10C, and NML, while “erasers” consists of ALKBH1, ALKBH3, ALKBH7, and FTO. “Readers” include m1A-binding proteins YTHDF1, YTHDF2, YTHDF3, and YTHDC1 (Li JX. et al., 2022). Currently, m1A has been shown to enhance the stability of mRNA.

### 2.5 ac4C

Previous studies on ac4C modification have focused on tRNA and rRNA. Acetylation affects ribosome maturation and protein translation efficiency, causing cell cycle abnormalities, inflammatory responses, cancer, and disease progression. Additionally, ac4C modification of mRNA has also emerged as a promoter of tumor progression (Feng et al., 2023). This change mainly occurs in the coding sequence (CDS) region of mRNA, which promotes mRNA structural stability and translation efficiency. Furthermore, ac4C affects mRNA translation in a position-dependent manner. Thus, the addition of ac4C modification to the Kozak sequence on the 5′UTR competitively inhibits translation initiation (Shi et al., 2023). The upstream open reading frame region of the 5′UTR, marked by rg4s (G-quadruplex) structure, inhibits CDS translation, prevents ribosome movement along the mRNA, and triggers the regulator of nonsense transcripts 1-mediated decay and degradation of mRNA (Jia et al., 2020). N-acetyltransferase 10 (NAT10)/Kre33, the only currently known protein with both an acetylase domain and an RNA-binding domain, catalyzes ac4C formation on RNA (Liao et al., 2023). NAT10 stabilizes the mRNA expression of *BCL9L*, *SOX4*, and *AKT1* through ac4C modification, which is involved in the malignant progression of bladder cancer (Wang et al., 2022). In addition, it mediates *NOTCH3* mRNA N4-acetylcytidine-dependent enhancement of mRNA stability, promoting tumor metastasis.

Therefore, NAT10-mediated ac4C modification plays a key role in various key pathways such as wingless-type MMTV integration site family (Wnt), transforming growth factor β, protein kinase B, and neurogenic locus notch homolog protein 1 signaling pathways, necessitating further clarification of its regulatory mechanisms. NAT10 is also linked to ferroptosis. It increases the mRNA stability of *ferroptosis inhibitor protein 1 (FSP1)* promoting the proliferation and metastasis of colon cancer cells. This mechanism represents a newly identified mechanism contributing to the development of colon cancer. Interestingly, *Helicobacter pylori* is a key factor affecting NAT10 expression, suggesting a novel avenue for exploring the involvement of pathogenic microorganisms in tumor progression (Deng et al., 2023).

### 2.6 Pseudouridine (Ψ)

Ψ, termed the “fifth nucleoside” of RNA, is the most prevalent nucleoside in various RNAs, affecting RNA stability, RNA-RNA/RNA-protein interactions, production of tRNA fragments (tRFs), splicing, and translation. The glycoside isomers of uridine involve C5, but not the heterocyclic atom N1, being bonded to the C1′ atom of pentasaccharide Ψ. In eukaryotes, the conversion of uridine to Ψ is catalyzed by a pseudouridine synthase (PUS or Ψ synthase), which acts as the “writer” of Ψ.

Six Ψ synthase families have been identified, including TruA, TruB, TruD, RsuA, RluA, and Pus10 (Rintala-Dempsey and Kothe, 2017). In eukaryotes, Ψ formation occurs through two main pathways. The first is RNA-independent, with PUS directly recognizing substrates and catalyzing the corresponding reactions (Nombela et al., 2021). The second pathway is RNA-dependent, where dyskerin, encoded by the DKC1 gene, binds to cassette H/ACA snRNA to perform the post-transcriptional modification. Dyskerin is also associated with human telomerase RNA containing H/ACA RNA motifs, potentially influencing cell senescence (Garus and Autexier, 2021). Additionally, an increased abundance of H/ACA snoRNA and DKC1 expression are involved in the progression of PCa (McMahon et al., 2015) (Table 1).

**TABLE 1:** Dynamic regulation process of prevalent RNA modifications and their impact on gene expression.

Modifications	Writers	Erasers	Readers	Major Functions
m6A	METTL3	FTO	FMRP	mRNA splicing
	METTL5	ALKBH5	IGF2BP1/2/3	mRNA stability
	METTL16	ALKBH3	YTHDF1/2/3	mRNA translation
	METTL14		YTHDC1/2	mRNA degradation
	KIAA1429			Chromatin regulation
	ZC3H13			
	WTAP			
	RBM15B			
	METTL3			
	ZCCHC4			
m7G	METTL1	Unknown	eIF4E	mRNA translation
	RNMT		CBC	mRNA Transcription extension
	WDR4			mRNA RNA stability
	WBSCR22			
m1A	TRMT6	ALKBH1	YTHDF1	mRNA RNA stability
	TRMT61A	ALKBH3	YTHDF2	
	TRMT61B	ALKHBH7	YTHDF3	
	TRMT10C			
	NML			
		FTO	YTHDC1	
m5C	NSUN2/5	TET1	YBX1	mRNA translation
			ALYREF	mRNA RNA stability
ac4C	NAT10	Unknown	Unknown	mRNA stability and translation efficiency
				Oocyte maturation
Ψ	TruA	Unknown	Unknown	Transcription extension mRNA stability
	TruB			mRNA stability; mRNA translation
	TruD			
	RsuA			
	RluA			
	PUS10			

**FIGURE 1:**
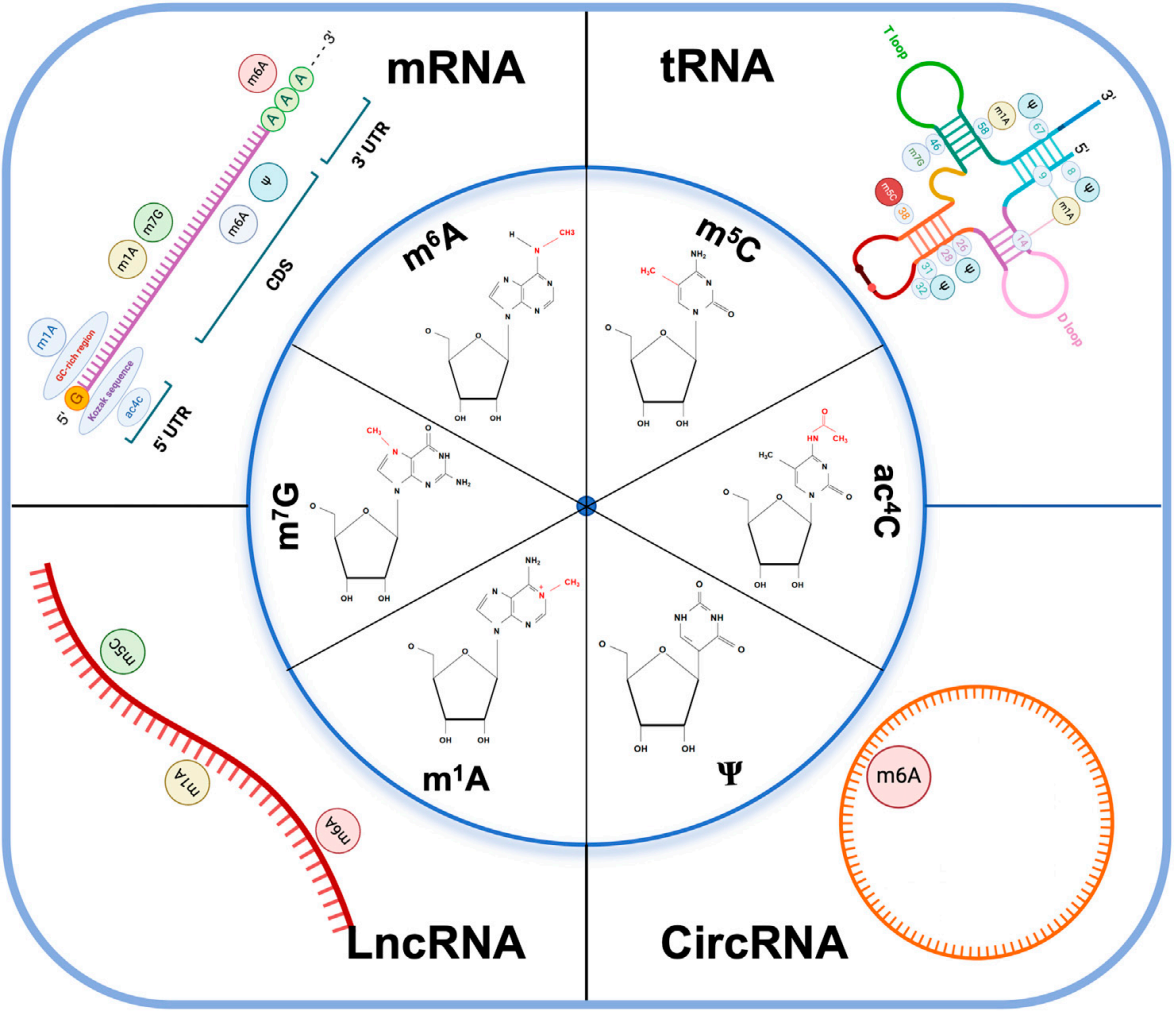
RNA modifications and their distributions on different RNA subtypes. The image includes the chemical structure of the six major types of RNA modification and the distribution of coding RNAs: mRNA, tRNA, and non-coding RNA: LncRNA, CircRNA. m6A N6-methyladenosine, m5C 5-methylcytosine, m1A N1-methyladenosine, m7G 7-methylguanosine, ac4C N4-acetylcytidine, ψ pseudouridine, CDS coding sequence, UTR untranslated regions. Kozak sequence: A nucleic acid sequence located behind the 5′end cap structure of eukaryotic mRNA, usually GCCACCAUGG, which can bind to translation initiation factors to mediate translation initiation of mRNA containing the 5′ cap structure. GC-rich region: GC content higher than 65%.

These modifications can alter RNA stability, splicing, translation, and localization, thereby affecting gene expression patterns and cellular processes implicated in cancer development. Meanwhile, these RNA modifications represent a complex layer of gene regulation that is increasingly recognized for its importance in cancer biology, offering potential targets for diagnostic and therapeutic interventions (Figure 1).

## 3 Significance of RNA modifications in PCa

### 3.1 RNA modification regulates malignant progression of PCa

#### 3.1.1 m6A

##### 3.1.1.1 METTL3

METTL3 directly regulates the expression levels of classical oncogenes or tumor suppressor genes, reflecting its important role in tumor progression. It also exerts methyltransferase activity, guiding the transcription of *MYC* mRNA and promoting the progression of PCa (Wan et al., 2023). Furthermore, the cancer-promoting factor METTL3 and its upstream cofactor YTH domain family 2 protein (YTHDF2), were identified as the key “writer” and “reader” of m6A modification, facilitating mRNA degradation of tumor suppressor lipid phosphatase and NK3 homeobox 1 and promoting the proliferation and migration of PCa cells.

This finding not only elucidates an additional pathway contributing to p53 signaling pathway inactivation beyond TP53 gene mutation or abnormal degradation of p53 protein but also reveals the key role of non-coding RNAs in PCa progression, providing a potential therapeutic target. Kinesin 3 (KIF3) is significantly more highly expressed in tumors than in adjacent tissues and may be involved in the malignant progression of PCa. *KIF3* mRNA undergoes m6A modification mediated by METTL3. m6A-activated *KIF3* mRNA promotes its stability by recruiting IGF2-binding protein 1 (IGF2BP1), representing a mechanism of its pro-cancer effect (Ma et al., 2021).

##### 3.1.1.2 YTHDF2

YTHDF2 is highly expressed in PCa and correlates with poor prognosis, while miR-493-3p inhibits its expression, restraining tumor malignancy (Li et al., 2020). The mRNA of *USP4* can bind to the RNA-binding protein HNRNPD, with its mRNA A2696 checkpoint modified by m6A via METTL3 and YTHDF2, resulting in mRNA degradation (Chen et al., 2021). Degraded USP4 inhibits ELAVL1 ubiquitination, decreasing the expression of Rho GDP-dissociation inhibitor 1, a risk factor for PCa metastasis and progression. METTL3 promotes m6A modification of *ITGB1* mRNA, affecting mRNA translation and promoting bone metastasis of PCa. Therefore, ELAVL1 may serve as a potential m6A modification target, warranting further exploration of its role beyond being an RNA-binding protein (Chen et al., 2021).

##### 3.1.1.3 WTAP

Wilms tumor-associated protein (WTAP), a splicing factor linked to WT1 protein, acts as a reader in m6A modification, forming a complex with METTL3 and METTL14, and mediates m6A modification in the 3′UTR regions of cirDDIT4 (Kong et al., 2023). m6A-modified circDDIT4 competitively combines with ELAVL1, downregulating the expression of anoctamin-7 and promoting the proliferation of PC cells.

##### 3.1.1.4 FTO

m6A demethylase FTO acts as a tumor suppressor in PCa. It promotes the stability of *chloride intracellular channel 4 (CLIC4)* mRNA through m6A modification, inhibiting the progression and metastasis of PCa (Zou et al., 2022). Furthermore, the expression of a non-coding RNA (FTO-IT1) located in the intron region of the FTO gene is significantly increased during PCa progression (Zhang J. et al., 2023). Mechanistically, the high expression of FTO-IT1 reduces m6A modification on mRNA by inhibiting the METTL3/METTL14/WTAP/RNA binding motif (RBM)15 methyltransferase complex and inhibits the stability and expression of p53.

#### 3.1.2 m5C

Cyclin-dependent kinase 13 (CDK13) regulates cell cycle progression and lipid metabolism-related gene transcription (Greifenberg et al., 2016). CDK13 expression is significantly overexpressed in PCa cells, where it induces fatty acid synthesis and promotes lipid deposition and tumor progression (Qi et al., 2021). Mechanistically, CDK13 mediates the Ser327 checkpoint phosphorylation modification of *NSUN5* (Zhang Y. et al., 2023), facilitating the m5C modification of *acetyl-* *CoA* *carboxylase 1* (*ACC1*) mRNA. The m5C-modified *ACC1* mRNA binds to ALYREF, resulting in its enhanced stability and nuclear export. Targeting m5C modification in the CDK13/NSUN5/ACC1 pathway may offer a new therapeutic approach for the treatment of PCa.

Nucleolar protein 2 (NOP2) is the only known methyltransferase that catalyzes m5C modification in PCa (Zhang H. et al., 2023). Elevated NOP2 expression has been considered a marker of poor prognosis, being associated with Gleason score, prostate-specific antigen (PSA) serum level, and recurrence after radical prostatectomy (Bantis et al., 2004). Mechanistically, lncRNA-LINC00963 competitively binds to miR-542-3p, activating the EMT signaling pathway and upregulating NOP2 levels (Sun et al., 2020). Additionally, NOP2 also catalyzes the methylation of cytoplasmic 28S rRNA and, as suggested by its expression in the late cell cycle G1 and S phases, it may also regulate the cell cycle (Bantis et al., 2004; Sharma et al., 2013).

#### 3.1.3 Ψ

Aberrant deposition of pseudoguanosine, dysregulated expression of pseudouridase, and somatic mutations have been associated with PCa (Taylor et al., 2010). PUS10, a pseudouridine synthase expressed in PCa nuclei, is involved in TNF-related apoptosis-inducing ligand (TRAIL)-induced apoptosis (Jana et al., 2017).

Mechanistically, PUS10 translocates into mitochondria, a process facilitated by Caspase-3 and chromosome region maintenance 1. It forms a positive regulatory loop with Caspase-3 and promotes the release of cytochrome c and mitochondrial pro-apoptotic protein (SMAC). Thus, SMAC mimetics may potentially serve as inhibitors in PCa treatment. However, the specific regulatory mechanism of Ψ modification in PCa requires further exploration.

### 3.2 RNA modification regulates the key signaling androgen receptor (AR) in PCa

PCa is closely related to androgen regulation, with the AR serving as a pivotal signaling molecule. AR plays a crucial role in elucidating the mechanisms underlying PCa occurrence, development, and drug resistance. Both ADT treatment in patients with hormone-sensitive PCa (HSPC) and AR pathway inhibitors (ARPI) treatment in patients with CRPC exert their effects by directly or indirectly affecting AR. AR amplification, expression of AR splice variants, and induction of AR coactivator expression are recognized as core mechanisms of drug resistance in PCa. However, the mechanisms underlying the RNA modification-mediated changes in AR expression need further clarification (Komiya et al., 2013).

The functional depletion of METTL3 in patients with PCa promotes the upregulation of AR and AR regulatory genes (Haigh et al., 2022). Furthermore, m6A regulators (METTL3, METTL14, WTAP, FTO, YTHDC1, YTHDC2, YTHDF1, YTHDF2, and YTHDF3) were significantly positively correlated with AR expression (Wu et al., 2021). METTL3 plays an important role in the variable shear of AR. These findings indicate the correlation between m6A and AR at the transcriptome level (Haigh et al., 2022). Additionally, Nie et al. revealed that m6A “reader” YTHDF1 knockdown reduces *AR* viability and expression in PCa cells by inhibiting the m6A methylation level of tripartite motif containing 68, a coactivator of *AR* (Nie et al., 2023).

Androgen receptor splice variant 7 (AR-v7) is the predominant AR mRNA splice variant in advanced PCa, contributing to drug resistance via ligand-independent persistent transcriptional activity. Demethylase ALKHB5-mediated m6A modification stabilizes the mRNA expression of the tumor suppressor gene *SIAH1*, inhibiting the production of oncogenic AR-v7 (Xia et al., 2022). Knocking down *SIAH1*, led to significantly increased expression of cleavage and polyadenylation specificity factor 1 (CPSF1). SIAH1 acts as an E3 ubiquitin ligase, directly binding to CPSF1 and mediating its ubiquitination-dependent degradation. Inhibition of CPSF1 reduces the binding capacity to the AAUAAA polyadenylation signal within the AR cryptic exon CE3, inhibiting the production of AR-v7. Further clarification is needed to elucidate the regulatory mechanisms involving RNA modification on *AR-* *v7* (Xia et al., 2022).

The key factor for RBPs YTHDF3 and G3BP1 to bind *AR* mRNA and regulate its localization and translation in a normal environment is METTL3-mediated m6A modification. “Reader” YTHDF3 binds m6A-modified *AR* mRNA with translational activity, while G3BP1 binds m6A-unmodified translation-inhibited *AR* mRNA. Under ARPI pressure, *AR* mRNA detaches from polysomes (PSs) and is recruited by RNA-protein stress granules (SGs), resulting in reduced translation of *AR*. These SGs promote the separation of m6A-modified or unmodified AR mRNA from its binding proteins YTHDF3 and G3BP1, protecting PCa tumor cells (Somasekharan et al., 2022).

**FIGURE 2:**
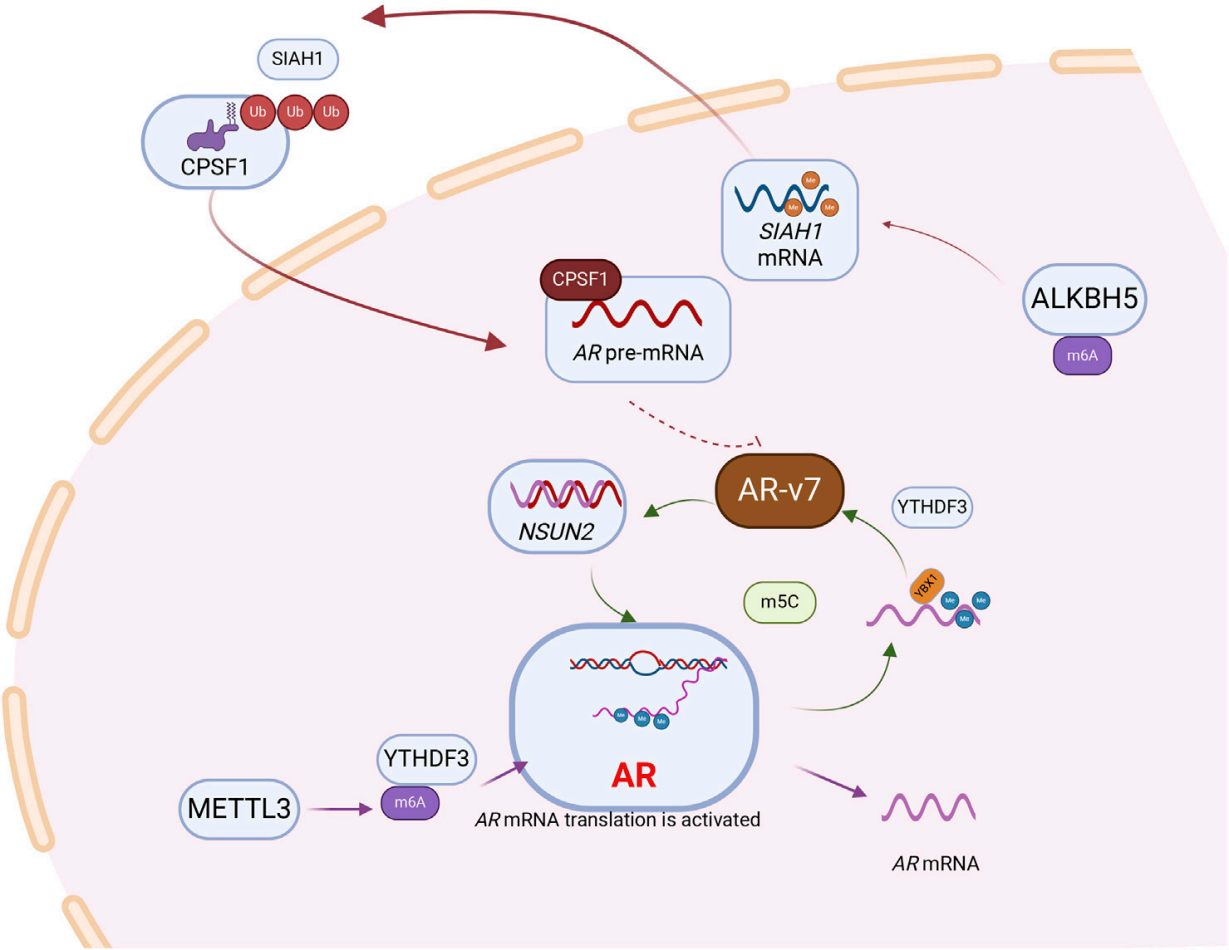
RNA modification is involved in the regulation of androgen receptor, a core signaling molecule in prostate cancer. Following the methylation of AR mRNA by the “Writer” METTL3, which mediates m6A modification, the “Reader” YTHDF3 recognizes and subsequently enhances the translational efficiency of AR mRNA.AR pre‐mRNA is modified by NSUN2 and recognized by YBX1 and maintains its stability. AR can act as a transcription factor to regulate the expression of NSUN2, and a positive feedback loop is formed between NSUN2 and AR to promote the progression of prostate cancer. Besides, NSUN2‐ m^5^C‐YBX1 axis could regulate AR-v7 expression levels. m6A modification mediated by demethylase ALKBH5 can stabilize the mRNA of SIAH1. SIAH1, as an E3 ubiquitin ligase, can degrade CPSF1, thereby further inhibiting AR-v7.

Most studies on RNA modifications affecting AR focus on m6A, with limited research on non-m6A modifications. m5C methyltransferase NSUN2 was shown to promote PCa progression by forming a positive feedback loop with ARs. Mechanistically, NSUN2 modifies *AR* mRNA with m5C, stabilized by YBX1. ARs also act as transcription factors, promoting the expression of NSUN2 and forming a positive feedback loop that promotes cancer. This suggests that targeting and reducing the expression of NUSN2 while using AR inhibitors may benefit PCa treatment (Zhu et al., 2022) (Figure 2).

### 3.3 RNA modifications in the tumor microenvironment of PCa

The tumor microenvironment substantially affects the malignant progression of cancer. The role of various immune cell types, cancer-associated fibroblasts, endothelial cells, stromal cells, and various other tissue-resident cell types in the tumor microenvironment has received increasing attention, as these components regulate tumor signaling pathways and molecules through direct or indirect cell-to-cell interactions, affecting tumor cell proliferation, metastasis, and drug resistance. However, our understanding of the extent of RNA modification of the components of the tumor microenvironment remains limited.

Bone metastasis is a critical event in PCa, often associated with poor prognosis and treatment failure. The intraosseous tumor microenvironment differs significantly from the primary tumor site, yet tumor cells quickly adapt and can even undergo secondary metastasis. Therefore, exploring the impact of the tumor microenvironment on bone metastasis and identifying reliable treatment targets is crucial. Bone metastasis microenvironment was shown to induce crown gene reprogramming and stem cell-like properties in tumor cells. Targeting RBM3 inhibits the expression of *CTNNB1* through METTL3-mediated mRNA methylation modification on its corresponding mRNA. Subsequently, it inhibits Wnt signaling, reducing stem cell induction in PCa cells by bone cells (Zhang SY. et al., 2023).

Due to the technical challenges and complex experimental verification required for RNA modification detection, along with the significant individual differences in the tumor microenvironment, some studies employ bioinformatics approaches. These approaches help explore the interaction between different RNA modifications within tumors and the tumor microenvironment, effectively mitigating the impact of individual differences. Zhai et al. constructed a model using data from The Cancer Genome Atlas (TCGA) and Gene Expression Omnibus public databases. They identified m7G-related differential genes (DEGs) and singled out NCBP2 and EIF4A1 as potential contributors to the malignant progression of PCa through m7G modification (Zhai et al., 2023). Similarly, Yu et al. developed a prediction model for m5C-related genes. They observed that NSUN2, TET3, and YBX1 expression levels increased with the advancement of PCa T stage. Furthermore, these three genes also served as predictors of patient survival. Further analysis of gene clusters exhibiting m5C differences revealed significant differences in the proportion of macrophages and CD8^+^ T cells, suggesting that m5C modification may participate in the regulation of immune cells within the tumor microenvironment and contribute to PCa risk by influencing immune infiltration (Yu et al., 2022).

Recently, immunotherapy has garnered increasing attention for its efficacy in treating solid tumors. In bladder and kidney cancer, immunotherapy has emerged as the first-line treatment (Lenis et al., 2020; Rizzo et al., 2022). However, PCa, characterized as a “cold tumor”, has often been regarded as an “immune desert” (Mulvey et al., 2022), with only 2%–3% of patients showing sensitivity to immunotherapy (Cornford et al., 2021). HNRNPA2B1 and METTL3, key regulators of m6A modification, play a significant role in immune regulation within the tumor microenvironment. Expression levels of HNRNPA2B1 and METTL3 were significantly lower in the high-immune PCa group than in the low-immune group (Jiang M. et al., 2020). Analysis of the TCGA database further confirmed a significant correlation between m6A regulators and the level of immune infiltration. Moreover, Xin et al. constructed a risk model comprising methylation-related genes (MRG) including m6A, m1A, m5C, and m7G, for PCa. They observed that the MRG score was lower in immunosuppressed PCa compared to immune-activated PCa (Xin et al., 2022).

**FIGURE 3:**
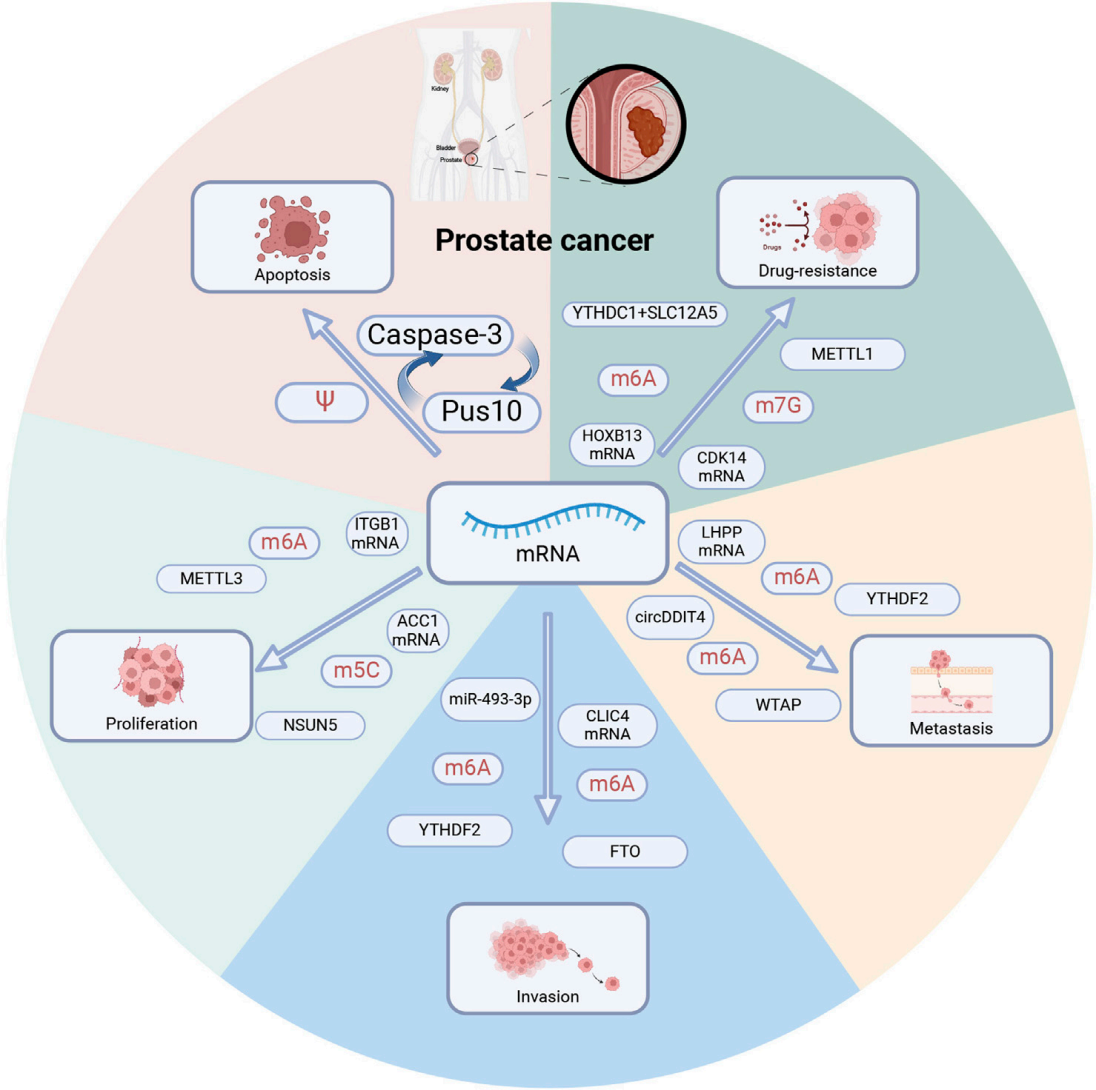
RNA modification regulates prostate cancer phenotype. Multiple RNA modification regulators regulate distinct mRNAs and contribute to tumor apoptosis, proliferation, invasion, metastasis, and drug resistance. PUS10: pseudouridine synthase. METTL1,METTL3: methyltransferase-like 1,3. YTHDC1, YTHDC2: YTH domain-containing 1,2. WTAP: Wilms tumor 1-related protein, a m6A methyltransferase. FTO: fat mass and obesity-associated protein, a m6A demethylase. NSUN5: m5C methyltransferase.

With the advancement of sequencing technologies, scRNA-seq has demonstrated tremendous potential for application. Heterogeneous nuclear ribonucleoprotein C (HNRNPC), a key m6A modification “Reader”, has been confirmed to be associated with tumor progression and immune suppression in lung cancer, liver cancer, and breast cancer (Lv et al., 2021; Cai et al., 2022). Li et al. used scRNA-seq to create a single-cell transcriptomic atlas of prostate cancer and found that HNRNPC-positive cells were significantly negatively correlated with T cells (Cheng et al., 2023). Furthermore, based on the differential expression of HNRNPC in single-cell data, it was found that the proportion of Treg cells in HNRNPC-positive cells was much higher than in negative cells. This suggests that HNRNPC may promote the activation of Treg cells and suppress effector CD8+ T cells through m6A modification. Similarly, Cai et al. established an m6A modification mRNA (MMM) score and analyzed single-cell data from 14 cases of prostate cancer to determine that the MMM score in the immune-enriched and nonfibrotic (IE) of TME subtypes was significantly higher than in the desert (D), fibrotic (F), and immune-enriched and fibrotic (IE/F) (Lu et al., 2023). However, the specific molecular mechanisms of these results still need to be explored. However, further exploration is needed to elucidate the specific molecular mechanisms by which PCa methylation regulators modulate the tumor microenvironment and to identify potential drug targets (Figure 3; Table 2).

**TABLE 2:** The regulatory relationship and biological role of RNA modification in prostate cancer.

RNA modification type	RNA modification regulatory factors	Expression in prostate tumor	Target	Function in prostate cancer
m6A	METTL3	High	*MYC*, *USP4*, *ITGB1*, *KIF3*, *ELAVL1*	Promoting tumor proliferation/metastasis/invasion
m6A	YTHDF2+METTL3	High	*LHPP/NKX3-1*	Promoting tumor proliferation/metastasis
m6A	YTHDF2	Low	*miR-493-3p*	Inhibiting tumor proliferation
m6A	WTAP	Low	*CirDDIT4*	Inhibiting tumor proliferation
m6A	FTO	Unknown	*CLIC4*	Inhibiting tumor metastasis/invasion
m6A	METTL3	High	*KIF3*	Promoting tumor proliferation
m6A	YTHDC1	High	*HOXB13*	Promoting tumor drug resistance
m6A	RBM15	High	*TPM1*	Promoting tumor drug resistance
m6A	YTHDF1	High	*TRIM68*	Inhibiting AR expression
m6A	ALKBH5	Low	*SIAH1*	Inhibiting AR-V7
m6A	YTHDF3, METTL3	High	*AR*	Promoting tumor drug resistance
m6A	METTL3+RBM3	High	*CTNNB1*	Regulating Wnt signaling pathway
m5C	NSUN5	High	*ACC1*	Promoting lipid deposition and tumor progression
m5C	NOP2	High	*lncRNA-LINC00963*	Regulating cell cycle and promoting tumor progression
m5C	NSUN2	High	*AR*	Promoting tumor progression
m7G	EIF4A1, NCBP2	High	*Unknown*	Promoting tumor proliferation
m7G	METTL1	High	*CDK14*	Promoting tumor drug resistance
Ψ	PUS10	High	*Caspase-3*	Promoting cell apoptosis

### 3.4 RNA modifications in drug resistance of PCa

The therapy for CRPC has been substantially challenging. METTL1 contributes to the progression of CSPC to CRPC. Specificity protein 1 promotes the transcription of METTL1 by recruiting P300 to bind to the METTL1 promoter region. High METTL1 expression m7G-modifies downstream *CDK14* mRNA stabilizing its expression and potentially driving CRPC progression (Zhang MP. et al., 2023).

The potassium-chloride cotransporter SLC12A5 is highly expressed in PCa, including in CRPC and neuroendocrine prostate cancer. It binds to YTHDC1 forming a complex and stabilizing its expression by m6A modification of the *HOXB13* mRNA. This is possibly one mechanism underlying PCa drug resistance (Yuan et al., 2023).

Ets-like transcription factor (ELK)-1 significantly influences PCa cell activities, including growth and differentiation. ELK1 overexpression enhances the YTHDF1-mediated m6A modification of *polo-* *like* *kinase 1 (PLK1)* mRNA, improving translation efficiency and leading to PCa progression (Li PZ. et al., 2022). Low expression of AZGP1P2, a pseudogene of AZGP1, correlates with poor prognosis. Our team explored its role in docetaxel sensitivity in CRPC for the first time. We found that AZGP1P2 was downregulated in PCa and stem-like tumor cell Mechanistically, AZGP1P2 combines with RBM15 and ubiquitin-like modifier activating enzyme 1, promoting the ubiquitination and degradation of the former at the protein level. Degraded RBM15 enhances the stability of *TPM1* mRNA in an m6A-dependent manner, contributing to tumor resistance acquisition (Wang et al., 2023).

## 4 The clinical application of RNA modification

As research progresses, increasing evidence suggests that RNA modification-related regulators may serve as relevant biomarkers for diagnosing and prognosing PCa (Lu et al., 2023). For example, Ji et al. identified the METTL family, IGF2BP3, and HNRNPA2B1 as significantly associated with the prognosis of PCa (Lang et al., 2021). Several scoring systems based on RNA modification-related regulators have demonstrated good predictive ability for patient recurrence, metastasis, and prognosis. Additionally, ψ modification, an important type of RNA modification, and its metabolite ψ are frequently detected in the urine and saliva of patients with various tumors such as colon and ovarian cancer. PCa cell lines (PC3, DU145) and tumor tissues exhibited higher levels of ψ than normal PCa epithelial cell lines (RWPE) and adjacent tissues, respectively (Stockert et al., 2019). Concomitantly, an elevated Ψ expression was found in the blood and urine of patients with PCa, suggesting its potential as a biological predictor besides PSA (Pérez-Rambla et al., 2017). Some small nucleolar RNAs (H/ACA snoRNA) and the protein isokeratin (DKC1), responsible for converting uridine to Ψ, are elevated in PCa tissues, potentially linked to the progression of PCa (Stockert et al., 2021). In recent years, mRNA vaccines have gained increasing attention as a promising treatment for tumors, emerging as a forefront therapy in future anti-tumor strategies (Lorentzen et al., 2022). The structural properties of mRNA functional domains often determine the efficacy of these vaccines (Orlandini von Niessen et al., 2019).

RNA modifications, such as m1A, m5C, and Ψ modifications, have great clinical application value in reducing the innate immunogenicity and molecular instability of mRNA vaccines. Incorporating mC5/Ψ and m1/Ψ into mRNA enhances its translation by 44-fold (Andries et al., 2015). An adjuvant-pulsed mRNA vaccine, utilizing m5C and Ψ modifications to enhance the delivery of resiquimod (C16-R848), significantly promotes the recruitment of CD8+ T cells in prostate tumor areas, enhancing the anti-tumor efficacy of mRNA vaccine-driven immune cells (Islam et al., 2021). Animal experiments have confirmed the efficacy of siARvm in alleviating tumor progression and improving survival rates in tumor-bearing mice, offering a promising new target for the treatment of advanced CRPC. Additionally, STM2457 and UZH2, two targeted inhibitors of METTL3, reduce m6A methylation levels and activity in both *in vivo* and *in vitro* experiments (Yankova et al., 2021). Several PCa cell lines have also shown sensitivity to these inhibitors, suggesting their potential for clinical validation pending a comprehensive assessment of biological safety and efficacy.

## 5 Conclusion

In summary, RNA modification, an important post-transcriptional regulatory mechanism, has garnered extensive attention over the years, with ample evidence highlighting its unique role in tumorigenesis and cancer progression. Specifically, RNA modifications profoundly influence signaling pathways and chromatin landscapes in tumor cells by affecting the stability of mRNA and the binding checkpoints of non-coding RNA. First, abnormal RNA modification closely affects tumor progression and prognosis. High-throughput screening of abnormal expression of RNA modification regulatory molecules in prostate cancer is beneficial to provide potential therapeutic targets and biomarkers, and provide personalized treatment measures for patients. Second, the molecular mechanisms of prostate cancer proliferation, differentiation and drug resistance are still unclear. Exploring the regulation of various RNA modification molecules on downstream proteins and pathways is crucial to clarify the mechanism of tumor progression. Third, AR is one of the most important targets for drug therapy. However, in different types of prostate cancer, such as hormone-sensitive prostate cancer and CRPC, there are qualitative differences in their expression abnormalities and the appearance of AR-v7 variant shear bodies. Exploring the role of RNA modification in it will complement the puzzle missing from classical genetics. Finally, integrating classical genomics, epitranscriptomics, proteomics, and bioinformatics to find key molecules affecting RNA modification in a multidisciplinary way and develop targeted drugs will provide optimism for potential transformative outcomes.
